# Cases of Eastern equine encephalitis in humans associated with *Aedes canadensis*, *Coquillettidia perturbans* and *Culiseta melanura* mosquitoes with the virus in New York State from 1971 to 2012 by analysis of aggregated published data

**DOI:** 10.1017/S0950268820000308

**Published:** 2020-04-01

**Authors:** J. A. Sherwood, S. V. Stehman, J. J. Howard, J. Oliver

**Affiliations:** 1 Department of Health, Central New York Regional Office, State of New York, Syracuse, New York 13202, USA; 2 Department of Forest and Natural Resources Management, College of Environmental Science and Forestry, State University of New York, Syracuse, New York 13210, USA; 3 Vector Surveillance Unit, Bureau of Communicable Diseases, Division of Epidemiology, Department of Health, Central New York Regional Office, State of New York, Syracuse, New York 13202, USA; 4 Division of Environmental and Renewable Resources, School of Agriculture Business and Technology, State University of New York, Morrisville, New York 13408, USA

**Keywords:** *Aedes*, Eastern equine encephalitis virus, human, mosquitoes, vector

## Abstract

From 1971 to 2012, in New York State, years with human Eastern equine encephalitis (EEE) were more strongly associated with the presence of *Aedes canadensis*, *Coquillettidia perturbans* and *Culiseta melanura* mosquitoes infected with the EEE virus (Fisher's exact test, one-sided *P* = 0.005, 0.03, 0.03) than with *Culiseta morsitans*, *Aedes vexans*, *Culex pipiens-restuans*, *Anopheles quadrimaculatus* or *Anopheles punctipennis* (*P* = 0.05, 0.40, 0.33, 1.00, 1.00). The estimated relative risk of a case in a year in which the virus was detected *vs.* not detected was 14.67 for *Ae. canadensis*, 6.38 for *Cq. perturbans* and 5.50 for *Cs. morsitans*. In all 5 years with a case, *Cs. melanura* with the virus was detected. In no year was there a case in the absence of *Cs. melanura* with the virus. There were 18 years with no case in the presence of *Cs. melanura* with the virus. Such observations may identify the time of increased risk, and when the methods may be used to prevent or reduce exposure to vector mosquito species in this geographic region.

## Introduction

Eastern equine encephalitis (EEE) neuro-invasive disease can be severe, with symptoms including seizures, paralysis, coma and death [1]. EEE has a mortality rate of 42% [2]. In the USA, the first recognised human cases occurred in the State of Massachusetts, in 1938 [3]. From 1964 to 2010, cases occurred in 17 States, and from 2009 to 2018, 72 cases occurred in 21 States [4]. The causative microorganism is the EEE virus. No specific antiviral treatment has been available [4]. A vaccine has been developed but has not been approved by the US Food and Drug Administration for general human use [4]. There is a potential risk of EEE in the European Union via importation of live animals carrying the virus, from North America [5]. A vaccine to prevent EEE has been commercially available for livestock.

The mosquito species *Culiseta melanura* (Coquillett) is a vector that maintains a life cycle of the virus, through birds [6, 7]. In New York State, in this study area, this species of mosquito breeds in a habitat of wooded wetlands [8]. Such habitats are scattered throughout and have been mapped [9]. The two largest are 16 and 20 km^2^.

In 1959, occasional testing of mosquitoes for arboviruses, including the EEE virus, began on Long Island in New York State [10], in the northeastern USA. The first reported case of EEE in a human in New York State [11] occurred in Oswego County in 1971, and within 1 week, surveillance for the EEE virus in mosquitoes began in Oswego County, and has continued in varying numbers of Counties annually until the present [12–14]. From 1971 to 2012, there were six cases diagnosed in humans in New York State [1, 15].

Occurrences of human cases [1, 9, 11, 13, 15] along with the presence of mosquito species having the virus [8, 13, 14, 16] have had a notable intermittency. Various mosquito species have been suggested as the transmitters of the virus to humans, or causative vectors [8, 16–23]. The objective of this work was to look for associations between cases in humans and the virus in mosquito species, towards identifying mosquito species that transmit the virus to humans.

## Methods

Preferred Reporting Items for Systematic Reviews and Meta-Analyses (PRISMA) guidelines were followed [24]. Data on human cases of EEE and on mosquitoes with the EEE virus were obtained from a US National Library of Medicine database, using search words: Eastern equine encephalitis, Eastern equine encephalitis virus, human, mosquito and New York State (http://www.ncbi.nlm.nih.gov/pubmed/). These data were originally obtained by the members of this Department of Health. One of the authors has been present since the beginning of data collection.

### Data

The study area consisted of the four contiguous counties of Madison (1710 km^2^), Oneida (3140 km^2^), Onondaga (2020 km^2^) and Oswego (3398 km^2^), located in a central geographic region of New York State having a centre point at a coordinate of 43.2° north and 75.8° west. Cases of EEE in humans and horses have been documented within a 3600 km^2^ area in these counties [15]. The study area has two wooded wetlands of 1600 hectares (16 km^2^) and 2000 hectares (20 km^2^) [8]. The environment, including geography, vegetation and animals, of this area has been described [8].

#### Cases

Cases of EEE in humans in New York State were obtained from published literature. For the study period 1971–2012, published articles with human cases covered the years: 1966–1977 [25], 1971 [11], 1970–1992 [13], 1971–2012 [1, 15], 1972–1974 [26], 1976 [27], 1978–1985 [9], 1990–1991 [28] and 1992–2012 [1, 15]. Data included dates of onset of cases in humans [1, 9, 15]. Dates of transmission were obtained from published cases or estimated based on an average of the incubation time period [1, 29]. The first five cases were located within an area that extended approximately 23 km north to south and 30 km east to west, all within the counties of Onondaga and Oswego. The distance between case 1 during 1971 and the western edge of a wooded wetland 16 km^2^ in Oswego County named ‘Toad Harbor’ was approximately 3 km [9]. The distance between case 2 during 1983 and the eastern edge of a wooded wetland 20 km^2^ in Onondaga County named ‘Cicero’ was approximately 0.8 km [9]. Cases 3–6 [15] were located approximately 6, 8, 3 and 29 km, respectively, from the closest large wooded wetland.

#### Mosquitoes

Detection of the EEE virus in mosquitoes in New York State was obtained from published literature. For the study period 1971–2012, published articles of surveillance for the EEE virus in mosquitoes covered the years: 1971 [11], 1970–1992 [13], 1972–1974 [26], 1976 [27], 1976–1977 [30], 1978–1985 [9], 1984–1991 [28], 1993–2012 [14] and 1994 [31]. Data included dates of collections of mosquitoes with the virus. A majority of the trapping sites were within an area of 2600 km^2^ which has had human and other vertebrate cases. Some trapping sites were located at the perimeters of the two largest wooded wetlands [28]. Among all mosquito poolings having the EEE virus, from 1971 to 2012, 80% were collected within 2 km of a wooded wetland [9]. The population and vector potential of a mosquito species, *Cs. melanura*, in these wetlands have been characterised in detail [16]. In these 42 years, there have been 11 species of mosquitoes in which the EEE virus was detected [14]. For this study, we did not include *Culex salinarius* Coquillett and *Psorophora ferox* (Humboldt). Standard nomenclature for mosquito genus and species was used [32–34]. The standard nomenclature of *Aedes canadensis* (Theobald) was used, in preference to a recently proposed renaming to *Ochlerotatus canadensis* (Theobald) [35].

### Statistical analysis

The hypothesis that the presence of an infected species of mosquito was associated with the presence of a case in a human was evaluated using Fisher's exact test [36]. This test was applied to 2 × 2 tables of the numbers of years with and without the EEE virus in each species of mosquito and the numbers of years with and without EEE in humans, using data in Table 1. A one-sided *P* value was calculated.

**Table 1.** Human cases of Eastern equine encephalitis disease and poolings of mosquitoes testing positive for the Eastern equine encephalitis virus, in the four counties of Madison, Oneida, Onondaga and Oswego, in Central New York State, 1971–2012^a^

Year	Cases of EEE	*Cs. melanura*	*Cq. perturbans*	*Cs. morsitans*	*Ae. canadensis*	*Ae. vexans*	*Cx. pipiens-restuans*	*An. quadrimaculatus*	*An. punctipennis*
1971	1^b,^^c^	1^d,^^e^	0^d,^^e^	3^d,^^e^	0^d,^^e^	0^d,^^e^	1^d,^^e,^^f^	0^d,^^e^	0^d,^^e^
1972	0	0	0	0	0	0	0	0	0
1973	0	0	0	0	0	0	0	0	0
1974	0	5	0	0	2	0	1	0	0
1975	0	0	0	0	0	0	0	0	0
1976	0	8	1	0	0	0	0	0	0
1977	0	20	0	1	0	0	0	*n*^g^	0
1978	0	1	0	0	0	0	0	0	0
1979	0	0	0	0	0	0	0	0	0
1980	0	2	0	0	0	0	0	0	0
1981	0	0	0	0	0	0	0	*n*	*n*
1982	0	0	0	0	0	0	0	*n*	0
1983	1	13	0	7	3	0	0	*n*	0
1984	0	0	0	0	0	0	*n*	*n*	*n*
1985	0	0	0	0	0	0	0	0	0
1986	0	0	0	0	0	0	0	0	0
1987	0	1	0	0	0	0	0	0	0
1988	0	2	0	1	0	0	0	0	0
1989	0	0	0	0	0	0	0	0	0
1990	0	73	2	5	3	2	0	1	0
1991	0	30	8	1	1	0	0	0	0
1992	0	0	0	0	0	0	0	0	0
1993	0	0	0	0	0	0	0	0	0
1994	0	19	0	0	0	0	*n*	0	0
1995	0	0	0	0	0	0	*n*	0	*n*
1996	0	2	0	1	0	0	*n*	*n*	*n*
1997	0	0	0	0	0	0	*n*	*n*	*n*
1998	0	0	0	0	0	0	*n*	*n*	*n*
1999	0	0	0	0	0	0	0	*n*	*n*
2000	0	0	0	0	0	0	0	*n*	*n*
2001	0	0	0	0	0	0	0	*n*	*n*
2002	0	0	0	0	0	0	0	*n*	*n*
2003	0	8	0	0	1	0	0	*n*	*n*
2004	0	11	1	0	1	1	0	*n*	*n*
2005	0	4	1	0	0	0	0	*n*	*n*
2006	0	66	0	0	0	0	1	*n*	*n*
2007	0	19	0	1	0	0	0	0	0
2008	0	16	0	0	0	1	0	*n*	1
2009	1	52	6	0	1	0	0	0	0
2010	2	61	4	0	1	0	0	0	0
2011	1	46	3	1	1	1	0	0	0
2012	0	1	0	0	0	0	0	0	0
Total of human cases	6								
Total of mosquito poolings		461	26	21	14	5	3	1	1

a An oversight in tabulating resulted in the change from ‘not tested’ to ‘tested’ for mosquito species *Cx. pipiens-restuans*, *An. quadrimaculatus*, *An. punctipennis* in years 2007–2011, in comparison with the analogous Table 3 in reference [14].

b Positive integer in this column denotes the number of human cases of EEE disease reported.

c ‘0’ in this column denotes no human case of EEE disease was reported.

d Positive integer in this column denotes the number of poolings of this mosquito species in which the EEE virus was found. Poolings were groupings of from 10 to 100 mosquitoes. The total number of poolings in which the virus was detected was 532.

e ‘0’ in this column denotes this mosquito species was found in collections and tested and the virus was not detected.

f *Culex pipiens* and *Culex restuans* were on occasion submitted as *Culex pipiens-restuans* group.

g ‘*n*’ denotes this mosquito species was not tested for the EEE virus.

An alternative hypothesis tested was that the proportion of years with human cases would be higher when the virus was present in a species of mosquito than the corresponding proportion of years with human cases when the virus was absent in a species of mosquito. Relative risk was used to quantify the strength of the association between years in which a human case was present with the presence of the virus in the mosquito species. Relative risk was defined as the probability of a human case occurring in a year in which the virus was present for the listed species divided by the probability of a human case occurring in a year in which the virus was absent for the listed species.

Statistical Analysis Software (SAS) was used (Cary, North Carolina, USA, http://www.sas.com).

## Results

From 1971 to 2012, there were six human cases of EEE and eight mosquito species for analysis (Table 1).

A statistical association, between the years with or without human cases and the years with or without the presence of the mosquito species with the virus, was found for *Ae. canadensis* (*P* = 0.005, Fisher's exact test), *Coquillettidia perturbans* (Walker) (*P* = 0.03) and *Cs. melanura* (*P* = 0.03) (Table 2). There was insufficient evidence to establish a statistical association between the number of years with or without human cases and the number of years with or without the presence of the virus in mosquito species *Culiseta morsitans* (Theobald) (*P* = 0.05), *Aedes vexans* (Meigen) (*P* = 0.40), *Culex pipiens-restuans* complex of *Culex pipiens* Linnaeus and *Culex restuans* Theobald (*P* = 0.33), *Anopheles quadrimaculatus* Say (*P* = 1.00) or *Anopheles punctipennis* (Say) (*P* = 1.00, Fisher's exact test) (Table 2).

**Table 2.** Estimated relative risk of the presence of human cases of Eastern equine encephalitis as a function of the presence or absence of mosquitoes testing positive for the Eastern equine encephalitis virus, in the four counties of Madison, Oneida, Onondaga and Oswego, in Central New York State, 1971–2012^a^

Species	Relative risk^b,^^c^	95% Confidence interval^d^	Proportion of human EEE when mosquito EEE virus present	Proportion of human EEE when mosquito EEE virus absent	Fisher's exact test, one-sided *P*-value
*Aedes canadensis*	14.67	1.86–115.49	0.444	0.030	0.005
*Coquillettidia perturbans*	6.38	1.27–32.05	0.375	0.059	0.03
*Culiseta morsitans*	5.50	1.08–28.08	0.333	0.061	0.05
*Culiseta melanura*	–	–	0.217	0.000	0.03
*Culex pipiens-restuans*	2.75	0.43–17.41	0.333	0.121	0.33
*Aedes vexans*	2.38	0.34–16.43	0.250	0.105	0.40
*Anopheles quadrimaculatus*	0.00	–	0.000	0.167	1.00
*Anopheles punctipennis*	0.00	–	0.000	0.185	1.00

a Using data in Table 1.

b Relative risk is defined as the probability of a human EEE case occurring in a year in which the EEE virus was present for the listed species divided by the probability of a human EEE case occurring in a year in which the EEE virus was absent for the listed species.

c Relative risk cannot be computed if the denominator is 0 (see *Cs. melanura*) and a confidence interval for relative risk cannot be computed if the proportion of human EEE when mosquito EEE virus present is 0.

d Confidence intervals that include a relative risk of 1 would not be statistically significant (*α* = 0.05) which is the case for the last four species listed in the table.

The estimated relative risk of a human case in a year in which the virus was detected *vs.* not detected, in a specific mosquito species, was 14.67 for *Ae. canadensis*, 6.38 for *Cq. perturbans* and 5.50 for *Cs. morsitans*. There was no year during which a case was found in a human when the virus was absent in *Cs. melanura* (Table 2).

### Temporal data

Dates, either documented or estimated, of virus transmission to human cases and dates, documented, of onset of symptoms in human cases were noted (Table 3). The dates of first and last detections of each mosquito species having the virus, and the number of days between these dates, were noted (Table 3), for context.

**Table 3.** Dates and time periods of detections of mosquito species having the Eastern equine encephalitis virus and occurrences of human cases, in the four counties of Madison, Oneida, Onondaga and Oswego, in Central New York State, 1971–2012

			Date^a^ of mosquito feeding on human case^b^	Date^a^ of onset of symptoms in human case	Date^a^ of first and last detection of mosquito species having virus					Time between first and last seasonal detection of mosquito having virus (days)	Time between first detection of mosquito having virus and mosquito feeding on human case (days)				
Year	Year (cardinal)	Case (ordinal)			*Cs. melanura*	*Cq. perturbans*	*Cs. morsitans*	*Ae. canadensis*	*Ae. vexans*	*Cs. melanura*	*Cs. melanura*	*Cq. perturbans*	*Cs. morsitans*	*Ae. canadensis*	*Ae. vexans*
1971	1	1	225	231	240–240	^c^	237–241	^d^	^c^	1	15	^c^	16	^d^	^c^
1983	12	2	229	237	193–279	^c^	193–252	206–221	^c^	87	36	^c^	23	23	^c^
2009	38	3	236	243	182–266	204–246	^c^	225–225	^c^	85	54	32	^c^	11	^c^
2010	39	4	213	220	181–266	210–236	^c^	236–236	^c^	86	32	3	^c^	23	^c^
2010	39	5	228	235	181–266	210–236	^c^	236–236	^c^	86	47	18	^c^	8	^c^
2011	40	6	208	215	199–272	202–230	234–234	230–230	250–250	74	9	6	22	22	42

a Julian date of the year (from 1 to 365).

b Based on incubation time periods documented for: 1971 case 1 of 5 days, and for 1983 case 2 of 8 days and for cases 3–6 estimated based on the average of (8 + 5)/2 = 7 days [1, 29].

c This mosquito species was found in collections and tested and the virus was not detected.

d This mosquito species was not found in collections.

### Spatial data

The cases of EEE occurred in two counties, Onondaga and Oswego. No cases occurred in the counties of Madison or Oneida. Mosquitoes with the virus were found in four counties, Madison, Oneida, Onondaga and Oswego. Of all mosquito poolings with the virus, approximately 91% were collected in the two counties of Onondaga and Oswego, from 1971 to 2012. The range of distances was 0.6–41.7 km, from human cases to trap sites with mosquitoes having the virus. Data on the distances between human cases and species of mosquitoes with the virus are shown in Table 4.

**Table 4.** Spatial relation between human cases of Eastern equine encephalitis and mosquito species testing positive for the Eastern equine encephalitis virus, in the four counties of Madison, Oneida, Onondaga and Oswego, in Central New York State, 1971–2012^a^,^b^

			Least distance between case and site of collections of mosquito species with virus (kilometres)				
Year	Year (cardinal)	Case (ordinal)	*Cs. melanura*	*Cq. perturbans*	*Cs. morsitans*	*Ae. canadensis*	*Ae. vexans*
1971	1	1	0.6	^c^	0.6	^d^	^c^
1983	12	2	5.2	^c^	5.2	5.2	^c^
2009	38	3	5.5	5.5	^c^	18.8	^c^
2010	39	4	6.9	2.7	^c^	21.0	^c^
2010	39	5	2.7	6.9	^c^	17.5	^c^
2011	40	6	10.7	13.2	23.3	41.7	41.7

a Among all mosquito poolings having the EEE virus, 80% were collected within approximately 2 km of a wooded wetland.

b A map of the study site has been published [9, 11].

c This mosquito species was found in collections and tested and the virus was not detected.

d This mosquito species was not found in collections.

## Discussion

Any of these species of mosquitoes having the virus has the potential to transmit the virus to a human [19, 20, 37]. In any human case, the actual mosquito that transmits the virus may depend on circumstances that include habitat, weather, flight of a mosquito, time of day or night and location of the host. In this study area, habitat and time period, the hypothesis that *Ae. canadensis*, *Cq. perturbans* and *Cs. melanura* transmitted the EEE virus to humans is supported by these statistical results. These species have been suspected as vectors [19, 20, 37]. It would be expected that species in which human blood has been detected would be the most likely candidates to transmit the virus.

The statistical association observed between cases in humans and the presence of *Ae. canadensis* with the virus is consistent with the previous results that human blood has been detected in wild caught *Ae. canadensis* [18, 19, 21]. *Ae. canadensis* hosts include mammals [18, 38]. *Ae. canadensis* feeds daytime [32, 38] and evening and early morning [32].

Similarly, the statistical association here between EEE in humans and the presence of *Cq. perturbans* infected with the EEE virus is consistent with the finding that human blood has been detected in field-collected *Cq. perturbans* [19] and that *Cq. perturbans* feeds on birds [17], on mammals [17] and on humans [32]. *Cq. perturbans* feeds daytime [33] and night-time [33, 38].

Field-collected *Cs. melanura* have been found to contain blood from humans [21], mammals [19, 21, 22] and birds [18, 19, 21, 22, 38]. A mix of mammalian and bird blood has been detected in *Cs. melanura* [19, 22]. The presence of both mammalian and bird blood in *Cs. melanura* is of epidemiological importance. It has been observed that *Cs. melanura* feeds more often on birds prior to mid-July [19] and feeds more often on mammals after mid-July, through to September [19]. This is consistent with a life cycle of the virus in birds maintained by *Cs. melanura* [6, 7, 9, 19, 39]. There were no human cases in any year without *Cs. melanura* having the virus (Tables 1 and 2). Cases in humans have been infrequent in the presence of *Cs. melanura* containing the virus [37, 40], despite *Cs. melanura* containing higher virus titres – on the order of 10^6^ plaque forming units per pooling of up to 50 mosquitoes [23]. In New York State, from 1971 to 2012, there were 5 years when there were human cases, and *Cs. melanura* with the virus were present, during those 5 years (Table 1); a similar feature was observed in a study of 26 years of data in Massachusetts, where *Cs. melanura* with the virus were present in 9 of 9 years with human cases [37]. There were 18 years when *Cs. melanura* with the virus was present, but during those 18 years, there were no human cases (Table 1); a similar feature was observed in that same study in Massachusetts, where *Cs. melanura* with the virus were present in 12 of 17 years without human cases [37]. In that same study in Massachusetts, there were 376 detections of the EEE virus in poolings of mosquitoes, of which 371 were *Cs. melanura*, four were *Cq. perturbans* and one was *Ae. canadensis* [37]; a similar feature was observed in New York State, where 563 detections of the EEE virus in poolings of mosquitoes, of which 461 poolings were *Cs. melanura*, 26 were *Cq. perturbans* and 14 were *Ae. canadensis*. During each of 4 years having human cases, 1983, 2009, 2010 and 2011, there were from 9 to 11 weeks during which *Cs. melanura* with the virus was present, but during these weeks, there were no additional human cases (Table 3). The persistent presence of *Cs. melanura* with the virus, from week to week, during the transmission season, has been shown in Massachusetts [40], similar to New York State.

This present analysis did not find a statistically significant association between human cases and the presence of *Cs. morsitans* with the virus. In a previous study, human blood was not detected in *Cs. morsitans* collected in the area of this present study [22]. *Cs. morsitans* with the virus has been present in some years with cases [9, 13], but not in other years [14, 15]. *Cs. morsitans* most often feeds on birds [19, 22], but will take blood from mammals [19, 22].

The results for *Ae. vexans* are anomalous. Human blood has been detected in field-collected *Ae. vexans* [18, 21], and *Ae. vexans* is attracted to humans [32] and mammals [17, 18, 32, 38], but there was no statistically significant association, here in this study, between human cases and the presence of *Ae. vexans* with the virus. This may have been due to the virus having been found at relatively low titres [41], on the order of 10^1^ plaque forming units per pooling of up to 50 mosquitoes [23]. EEE virus has not been detected in *Ae. vexans* saliva as shown by the absence of cytopathic effect on baby hamster kidney cell cultures [42].

The statistical results for *Cx. pipiens-restuans* complex of *Cx. pipiens* and *Cx. restuans*, *An. quadrimaculatus* and *An. punctipennis* may be because these three species had 6, 17 and 14 years, respectively, during which these species were not tested for the virus (Table 1). The inability to detect a statistically significant association for any of these three species could be due to the lack of statistical power. It is possible that none of these species has an association with the occurrence of human EEE. The host preferences of *Cx. pipiens* and *Cx. restuans* are for birds [18, 21, 38] although human blood has been detected in field-collected *Cx. pipiens* and *Cx. restuans* [21]. Human blood has been detected in field-collected *An. quadrimaculatu*s [21]. *An. quadrimaculatu*s saliva has been found to contain the EEE virus after feeding on viremic chicks, as saliva produced a cytopathic effect on baby hamster kidney cell cultures [42]. *An. punctipennis* saliva did not have the virus after feeding on viremic chicks [42]. Human blood has been detected in *An. punctipennis* [21].

There was a spatial relationship between human cases and mosquitoes with the virus. All cases were located in only two counties, and 91% (483 of 532) of mosquito poolings with the EEE virus were collected in those two counties. Flight distance maximums, for *Cs. melanura* of 9.1 km and for *Cs. morsitans* of 9.8 km, have been determined [43]. A flight distance maximum for *Ae. vexans* of 48 km was determined [44]. Flight distances for *Cq. perturbans* and *Ae. canadensis* have been determined to be 5 and 2 km, respectively [38]. The longest distance between any human case and either of the two largest *Cs. melanura* breeding habitats, the two named wooded wetlands, was 29 km. Thus, distances between human cases and mosquito traps were within ranges published.

### Limitations of this study

This study has the limitation of being dependent on reporting of cases by doctors, hospitals, commercial laboratories, agency laboratories and departments of health of counties and cities [45, 46]. During the period from 1971 to 2012, there were or may have been changes in medical practice, clinical diagnosis, laboratory testing, insurance, finance, public health law, code, rules and regulations, definitions of cases, requirements for reporting, employee interpretations and agency feasance [45–49]. A delay or oversight in recognition, testing, tabulating or reporting may result in a subsequent addition or subtraction of an occasional human case or mosquito detection of the virus.

Sampling error can occur, because the sample sizes customarily allowed or accepted and published by laboratories have been 10–100 individual mosquitoes. If the prevalence of the virus in a population of a species of mosquito was one in 1 000 or one in 10 000, then nine of 10 samples, referred to here as poolings or pools, may have no virus detected. In such instances, the results may lead to a conclusion that virus was not present in that population, when in actuality it was present at a level below the limit of detection in that circumstance.

This study applies to one region of New York State and is not necessarily generalisable to other geographic areas of the state or country. The laboratory that tested for the virus in mosquitoes set limits to the number of poolings of mosquitoes that each county could send for testing. Each county made the decision on what species of mosquitoes to send for testing, based in part on the species of viruses of concern for public health or vector control. Therefore, not all species of mosquitoes could be sent to the laboratory for testing. The absence of a statistically significant association between the presence of cases in humans and the virus in *Cx. pipiens-restuans*, *Ae. vexans*, *An. quadrimaculatu*s or *An. punctipennis* should not be misinterpreted to imply that these species could not transmit the virus to humans in other circumstances.

### Practical application

This information may be useful for public health officials deciding whether or not to initiate methods to suppress vectors, for individuals using personal protection to reduce exposure to mosquitoes and for clinicians to include EEE in the differential diagnosis of encephalitis.
